# Comparison of the Virulence Potential of *Acinetobacter* Strains from Clinical and Environmental Sources

**DOI:** 10.1371/journal.pone.0037024

**Published:** 2012-05-24

**Authors:** Azam F. Tayabali, Kathy C. Nguyen, Philip S. Shwed, Jennifer Crosthwait, Gordon Coleman, Verner L. Seligy

**Affiliations:** Biotechnology Laboratory, Environmental Health Sciences and Research Bureau, Environmental Health Centre, Health Canada, Ottawa, Canada; Monash University, Australia

## Abstract

Several *Acinetobacter* strains have utility for biotechnology applications, yet some are opportunistic pathogens. We compared strains of seven *Acinetobacter* species (*baumannii*, Ab; *calcoaceticus*, Ac; *guillouiae*, Ag; *haemolyticus*, Ah; *lwoffii*, Al; *junii*, Aj; and *venetianus*, Av-RAG-1) for their potential virulence attributes, including proliferation in mammalian cell conditions, haemolytic/cytolytic activity, ability to elicit inflammatory signals, and antibiotic susceptibility. Only Ah grew at 10^2^ and 10^4^ bacteria/well in mammalian cell culture medium at 37°C. However, co-culture with colonic epithelial cells (HT29) improved growth of all bacterial strains, except Av-RAG-1. Cytotoxicity of Ab and Ah toward HT29 was at least double that of other test bacteria. These effects included bacterial adherence, loss of metabolism, substrate detachment, and cytolysis. Only Ab and Ah exhibited resistance to killing by macrophage-like J774A.1 cells. Haemolytic activity of Ah and Av-RAG-1 was strong, but undetectable for other strains. When killed with an antibiotic, Ab, Ah, Aj and Av-RAG-1 induced 3 to 9-fold elevated HT29 interleukin (IL)-8 levels. However, none of the strains altered levels of J774A.1 pro-inflammatory cytokines (IL-1β, IL-6 and tumor necrosis factor-α). Antibiotic susceptibility profiling showed that Ab, Ag and Aj were viable at low concentrations of some antibiotics. All strains were positive for virulence factor genes *ompA* and *epsA*, and negative for mutations in *gyrA* and *parC* genes that convey fluoroquinolone resistance. The data demonstrate that Av-RAG-1, Ag and Al lack some potentially harmful characteristics compared to other *Acinetobacter* strains tested, but the biotechnology candidate Av-RAG-1 should be scrutinized further prior to widespread use.

## Introduction

The genus *Acinetobacter* comprises 27 known species and several unnamed provisional species of gram-negative, ubiquitous, non-motile, non-fermentative, coccobacilli [1]–[2]. Many members share phenotypic features, but are not well defined with respect to beneficial and harmful characteristics [3].

In recent years, certain strains of different *Acinetobacter* species have been developed for bioremediation of recalcitrant and harmful organic chemicals, as well as bioengineering of enzymes and diagnostic materials [4]–[8]. One promising example is the RAG-1 strain of *A. venetianus*, which has been shown to produce several oil-modifying substances such as emulsans, esterases, lipases and surfactants [9]–[11]. This strain was previously assigned to other species groups (*A.calcoaceticus and A.lwoffi*) [12] which are recognized as opportunistic pathogens. Further advancement of the *Acinetobacter* genus for beneficial applications requires a rigorous clarification concerning threats to drinking water [13]–[14] and linkages to nosocomial infections [15]–[20].

Clinically, *A.baumannii* (Ab) is most often identified as the cause of infection, but others include *A.calcoaceticus* (Ac), *A.haemolyticus* (Ah), *A.lwoffii* (Al) and *A.junii* (Aj) [20]–[26]. The majority of reported clinical cases involved pneumonia/pulmonary infections and septicaemia, but others included endocarditis, meningitis, burn and surgical wound infections, and urinary tract infections. Many clinical isolates have acquired multiple antibiotic resistance [27] [17] and can be more pan-resistant than even methicillin-resistant *Staphylococcus aureus* (MRSA) [28]. Furthermore, infections caused by *Acinetobacter* are not restricted to the clinical setting, and reports have emerged describing cases involving otherwise healthy individuals of varying ages, occurring in community settings, during wars, and following natural disasters [29]–[36].

To date there has been no comparative testing for potential virulence and toxic effects of *Acinetobacter* species/strains isolated from clinical and environmental sources, which should be necessary prior to any intended biotechnology application. Our research objective is to expand the repertoire of useful endpoints required to predict and rank pathogenicity potential of environmental *Acinetobacter* strains with the aim of reducing the use of more costly and laborious *in vivo* test methods. In previous work, we developed a set of pathogenicity-toxicity parameters that could differentiate between potentially harmful and non-toxic strains of *Bacillus* species [37]–[39]. Here we describe a side-by-side comparison of strains from seven *Acinetobacter* strains which were selected from both clinical and environmental sources (See Table 1). We used our previous test parameters, as well as others developed to study clinical *Acinetobacter* strains. The latter include assays which assess capacity to bind or adhere to mammalian cells [40]–[43], disrupt mammalian cell interactions (attachments) [41]–[42] [44]–[49], produce haemolytic or cytolytic activities [41] [50]–[51], and/or infect by way of proliferation in mammalian cell culture medium. Also included is an assessment of exposure-induced immune system responses such as release of pro-inflammatory cytokines and chemoattractants (interleukin (IL)-1β, IL-6, IL-8, and tumour necrosis factor-α (TNF-α)), as observed *in vitro* with laryngeal epithelial cells [48] and cultured mouse splenocytes [52].

**Table 1:** *Acinetobacter* strains used in this study.

Bacterium	ATCC #	Abbrev.	Previous Genomospecies Designation	Biohazard Level*	Isolation Source of Strain as reported by ATCC
*A. baumannii*	9955	Ab	2	2	Human spinal fluid
*A. calcoaceticus*	23055	Ac	1	2	Soil (Type Strain)
*A. guillouiae*	11171	Ag	11	1	Sewage containing gas-works effluent (Type Strain)
*A. haemolyticus*	17906	Ah	4	2	Human sputum (Type Strain)
*A. junii*	17908	Aj	5	2	Human urine (Type Strain)
*A. lwoffii*	15309	Al	8/9	2	NA (Type Strain)
*A. venetianus*	31012	Av-RAG-1	NA	1	Tar on beach (Type Strain)

* Classified by ATCC according to U.S. Public Health Service guidelines.

NA – Not Available.

## Materials and Methods

### Bacterial Culture and Monitoring

Table 1 contains a list of the bacteria used in this study, along with their acquisition sources, species abbreviations, and biohazard ranking as designated by ATCC. Strains were selected based on availability from known repositories (ATCC), and quantity of literature information on the each strain. Almost all strains selected were Type Strains (except Ab ATCC# 9955), although this was not part of the selection criteria. In all experiments, level two biosafety facility and procedures were used. Bacterial stocks were adjusted to10^8^ cfu/mL, stored at −80°C and routinely checked for viability, morphology and homogeneity by plating on Luria-Bertani (LB)-agar. For assessment of Ag growth, nutrient broth (NB) plates were used, which permitted more rapid results compared to LB plates. Growth of bacteria was assessed at 28°C and 37°C with turbidity measurements (optical density at 450 nm (OD_450_)) over a 24 h period using an automated scanning multiwell spectrophotometer (Spectramax Plus 386, Molecular Devices Co., Sunnyvale CA). Additional monitoring for growth and viability of cultures was done by measuring bioreduction activity after incubating cultures with 0.5 mg/mL XTT (sodium 3′-{1-[(phenylamino)-carbonyl]-3,4-tetrazolium}-bis{4-methoxy-6-nitro} benzene sulphonic acid hydrate), as done previously with *Bacillus* organisms [53]. Bacterial reduction of the tetrazolium salt XTT to XTT formazan was measured at OD_480_ every 15 min.

Cell-free bacterial culture filtrates were prepared by inoculating 10 mL of nutrient broth with 10^8^ cfu of bacteria. Following 24 h at 28°C or 37°C, cultures were centrifuged at 12,000×G and the supernatants were filtered through a 0.22 µm syringe filter (Millipore Corp, Bedford, MA).

### Bacterial Antibiotic Susceptibility and Haemolytic Activity

The Minimal Inhibitory Concentration (MIC) assay was conducted as described by Seligy and Rancourt [54]. Antibiotics were purchased from the Sigma Chemical Company (Oakville, ON) and Invitrogen (Carlsbad, CA). Using 96-multi-well plates, 2×10^4^ cfu of bacteria were added to each microwell containing an antibiotic dilution series in trypticase soy broth (TSB) (200 µL final volume). The final concentration of each antibiotic in the series was 24, 12, 6, 3, 1.5, 0.75, 0.38 and zero µg/ml. Since growth in TSB varied between strains, plates were incubated at 28°C or 37°C for 24 h, 48 h or 96 h to generate enough bacterial growth to evaluate antibiotic susceptibility. The bacterial metabolic status of each sample was determined by adding MTT (3-(4,5-dimethylthiazol-2-yl)-2,5-diphenyl tetrazolium bromide) to a final concentration of one mg/ml/well. Plates were then incubated at 28°C or 37°C for two hours and examined for purple crystals. The MIC was defined as the minimum antibiotic concentration resulting in no detectable MTT bioreduction, which indicates that no metabolic activity and proliferation of bacterial cells has occurred.

Haemolytic activity produced by various *Acinetobacter* strains was compared to that produced by the positive control (*Bacillus cereus* ATCC# 14579). Sterile, defibrinated sheep blood (Cedarlane Laboratories, Hornby, Ontario) was washed three times with PBS by repeated centrifugation and resuspension in 0.9% (w/v) saline, before resuspension at a final concentration of 5% (v/v) with cooled (45°C), autoclaved agar (1.2% (w/v)) containing 1.4% (w/v) pancreatic digest of casein, 0.5% (w/v) NaCl, 0.45% (w/v) peptone and 0.45% (w/v) yeast extract. After agar solidification, each strain was deposited (10^3^ cfu/10 µL spot) in a grid pattern onto the surface, and incubated at 37°C for up to 6 days. Photographs were taken daily to monitor haemolytic progression.

### Mammalian Cell Culture and Exposures

Human HT29 colonic epithelial cells and mouse J774A.1 macrophage were obtained from the American Type Culture Collection (Rockville, MD). Cell monolayers were maintained at less than 80% confluence in mammalian cell culture medium (MCCM; Dulbecco's Modified Eagles Medium containing 25 mM glucose, 10% fetal bovine serum and 2 mM glutamine with/without 50 µg/mL gentamicin). Exposures were conducted by exposing mammalian cells to 10^2^ to 10^6^ cfu for zero to 24 h using 96-well cell culture plates (100 µL final). Some experiments designed to study the effects of bacterial protein products included the addition of a protease inhibitor cocktail (Complete™ Tablets; Roche Diagnotics, Penszberg, Germany) during *Acinetobacter* exposure.

The effects of bacterial exposure on mammalian cell morphology were monitored by fluorescence and confocal microscopy. Cell monolayers were pre-grown on glass coverslips, fixed for 5 min with 4% (w/v) paraformaldehyde in PBS (pH 7.4), stained with 0.25% (v/v) SYTOX™ green for 10 min, and for 5 min each with 100 µg/mL Texas Red-conjugated wheat germ agglutinin and rhodamine-phalloidin (1∶40), according to the manufacturers = procedures (Molecular Probes Inc, Eugene, OR). Results were viewed and photographed with a Nikon TE-2000 Eclipse microscope equipped with epifluorescence optics and either a Nikon Coolpix 990 digital camera or a Nikon C1 confocal with 488, 543 and 633 lasers.

Quantitative changes in metabolism of mammalian cells during bacterial exposure was monitored by measuring MTT bioreduction activity as described previously [37]–[38]. Following exposures, the culture supernatants were removed and processed for cytokine content as described in the next section. Fresh mammalian culture media supplemented with MTT at a final concentration of one mg/mL was added to the wells. The plates were incubated for two hours at 37°C and the wells were rinsed twice with PBS to remove non-adherent cells and bacteria. Following addition of 100 µL/well DMSO (Sigma), solubilized-formazan color change was measured at OD_505_. Bioreduction activity of exposed cells was expressed as percentage activity compared to control PBS-treated cells.

### Cytokine Measurements

Cytokine activity was measured using supernatants from individual exposures. Culture supernatants (100 µL) were transferred to membrane-containing 96-well (0.45 µm hydrophilic Multiscreen^7^ plates, Millipore Corp, Bedford, MA). The filtrates were collected through a vacuum manifold into empty 96-well plates. The bacteria and debris-free filtrates were frozen at −80°C until further analysis for cytokine content. After thawing, levels of IL-1β, IL-6, IL-8 and TNF-α were measured using a multiplex liquid bead array assay (BioRad, Laboratories Inc., Mississauga, ON), and validated using enzymatic immunosorbant assays [55] with the following types of antibodies: capture antibodies, human anti-IL-8 (4 µg/mL; R&D Systems, Minneapolis, MN), murine anti-IL-1β (4 µg/ml), anti-IL-6 (0.5 µg/ml), anti-TNF-α (0.5 µg/ml) (PeproTech, Rocky Hill, NJ); coating antibodies, biotinylated anti-human IL-8 (20 ng/mL; R&D Systems), biotinylated anti-murine IL-1β (100 ng/ml), anti-IL-6 (300 ng/ml), anti-TNF-α (300 ng/ml; PeproTech). Recombinant IL-8 (R&D Systems), IL-1β, IL-6, or TNF-α (PeproTech) were used as standards. Lipopolysaccharide (LPS; 0.1 to 1.0 ng/mL) from *Escherichia coli*, *Salmonella typhimurium* and *Serratia marcescens* (Sigma, Oakville, ON) was used as a positive control for cytokine expression studies.

### Macrophage Bactericidal Assay

Killing of bacteria by macrophage was tested using ∼80% confluent J774A.1 monolayers (∼10^5^ cells per 33 mm^2^ well) and 10^3^ bacteria in 100 µL of mammalian cell culture medium without antibiotic. Several incubation times were tested and optimal conditions were determined to be four hours at 37°C. Following incubation, the entire well contents were scraped, serially diluted in PBS and then plated on LB-agar. Colonies were enumerated over a period of 18 to 72 h at 37°C. Control experiments included wells with only bacteria, wells containing only J774A.1 with no bacteria, and bacteria with HT29 epithelial cells.

### PCR, Southern Hybridization and Sequencing

Primers for reported virulence factor genes (*epsA* and *ompA*; [40] [47] [56] and the quinolone resistance determining regions (QRDRs) of the constitutive genes *parC* and *gyrA*, where mutations may confer fluoroquinolone resistance [57]–[60], were designed from the *Acinetobacter baumannii* ATCC17978 genome. Primer sequences were cross referenced to other complete *Acinetobacter* genomes through Microbial Genomes Blast (http://www.ncbi.nlm.nih.gov/sutils/genom_table.cgi) to ensure primers were not strain specific (Table 2). Fragments were amplified from 100 ng of genomic DNA template using standard reaction conditions (AmpliTaq core reagents, ABI, Carlesbad, CA) with 0.6 µM of each primer and the following reaction parameters: 30 cycles 94°C, 15 sec; 50°C, 30 sec; 72°C, 1∶30. PCR reactions were fractionated by gel electrophoresis (1% agarose) and amplicons were purified using a gel extraction spin kit (Qiagen, Germantown, MD).

**Table 2:** PCR Primers Used in this Study and Resulting Amplicon Sizes.

Gene	Accession	Forward Primer	Reverse Primer	PCR Amplicon/ORF Size (bp)
*ompA*	AY485227	CGCTTCTGCTGGTGCTGAAT	CGTGCAGTAGCGTTAGGGTA	531/1317
*epsA*	NC_011595	AGCAAGTGGTTATCCAATCG	ACCAGACTCACCCATTACAT	451/1101
*gyrB*	CT025946.2	ATGAGCGTATCGGAAATCCG	ACGTCAACAATACCTGATTTACC	485/2713
*parC*	CU459141.1	GGCTGGTCTTCTTCACGAATA	CCTTGCGCATCATGCGACAG	581/2220

Genomic DNA (two µg) from each *Acinetobacter* strain was digested with 10 units of EcoRI (New England BioLabs, Pickering, ON) at 37°C for three hours followed by electrophoresis on (1% agarose gels, 60 V, four hours). DNA contents of gels were pre-treated (15 min acid depurination in 0.25 M HCl, 30 min denaturation in 0.5 M NaOH, 1.5 M NaCl, 30 min neutralization in 0.5 M NaCl, 1.5 M Tris HCl pH 7.5) before overnight transfer onto Nytran supercharge nylon membranes (Whatmann, Piscataway, NJ) in 20× SSC (3 M NaCl, 0.3 M sodium citrate, pH 7.0). Purified Ab *ompA* and *epsA* PCR products were labelled with ^32^P-dCTP using the Bioprime random labelling kit (GE Healthcare, Baie d'Urfe, QC) and hybridized overnight to membranes at 42°C in UltraHyb hybridization buffer (50% formamide) (Ambion, Austin, TX). Blots were washed twice with 2× SSC, 0.1% sodium dodecyl sulphate (SDS) for 10 min at 42°C then twice with 0.5× SSC, 0.1% SDS for 15 min at 65°C. Membranes were exposed to phosphorimager screens for two hours then scanned with the Typhoon scanner (GEHealthcare, Baie d'Urfe, QC).

Amplicons of *gyrA* and *parC* were sequenced using BigDyeTerminator v3.1 sequencing kit (Applied Biosystems, Streetsville, ON) and gene specific forward and reverse primers. Reactions were set up with 1/8^th^ reaction mix, sequencing buffer, 0.5 µM primer and 50 ng PCR template. Reactions were purified using Centri-Sep cycle sequencing clean up columns (Princeton Separations, Adelphia, NJ). Sequencing reactions were run on an Applied Biosystems 3130xl Genetic Analyzer on a 36 cm capillary array using POP-7™ polymer (Applied Biosystems, Streetsville, ON). Sequences were assembled and translated using VectorNTI v11 software (Invitrogen, Carlsbad, CA).

### Statistical Analysis

The significance of the differences in cytokines produced by mammalian cells in response to different *Acinetobacter* strains was done by comparing their levels with an analysis of variance (ANOVA) followed by a post-hoc Tukey Multiple Comparison Test. Statistical analyses were done with SigmaPlot software (Systat Software Inc, San Jose, CA).

## Results

### Microbial Growth and Influence of Mammalian Cells

The proliferation capacity of the different *Acinetobacter* strains was compared in a bacterial medium (LB) and mammalian cell culture medium at 37°C. The results summarized in Table 3 are derived from experiments using bioreduction of XTT as a sensitive measure of bacterial growth and viability. In LB medium, the majority of strains reduced XTT at low to high inoculation concentrations. In contrast, almost all strains failed to reduce XTT in mammalian cell culture medium at low seeding densities, aside from Ah, which readily grew in all test conditions. Bioreduction was improved with high inoculation concentrations, and when HT29 cells were present. The exception was Av-RAG-1, which was highly bioreductive compared to all other strains in LB, but showed the lowest bioreduction in mammalian cell culture medium and no bioreduction in the presence of HT29 cells. A similar assessment using macrophage-like J774A.1 cells was not possible due to killing of most strains (see Section on Macrophage-like J774A.1 Exposures).

**Table 3:** *Acinetobacter* Growth-related Bioreduction Activity.

Test Bacterium	Optical Density of XTT-Formazan Produced at 24 h*								
	LB			DMEM			DMEM+HT29		
	10^2^ cfu	10^4^ cfu	10^6^ cfu	10^2^ cfu	10^4^ cfu	10^6^ cfu	10^2^ cfu	10^4^ cfu	10^6^ cfu
**Ab**	0.40±0.25	2.3±0.1	>3.2†	0.00	0.00	1.14±0.02	0.00	1.13±0.1	2.42±0.19
**Ac**	0.01±0.02	0.01±0.01	0.67±0.13	0.00	0.00	0.4±0.04	0.11±0.01	0.26±0.01	2.28±0.27
**Ag**	0.00	0.03±0.02	0.89±0.05	0.00	0.00	0.12±0.03	0.18±0.03	0.23±0.02	0.33±0.14
**Ah**	1.50±0.11	1.73±0.16	2.18±0.20	0.12±0.08	1.28±0.14	1.63±0.14	0.64±0.08	0.92±0.11	1.43±0.15
**Aj**	0.04±0.06	0.03±0.05	2.95±0.14	0.00	0.00	1.47±0.17	0.08±0.01	0.42±0.07	1.57±0.16
**Al**	0.00	0.03±0.02	1.56±0.13	0.00	0.00	1.14±0.15	0.21±0.02	0.30±0.05	1.56±0.13
**RAG-1**	1.85±0.08	2.39±0.06	2.92±0.06	0.00	0.00	0.53±0.04	0.00	0.00	0.00

* Data represent means ± standard deviation from six replicates.

† Upper Detection Limit of plate reader.

### Haemolytic Activity

**Figure 1:**
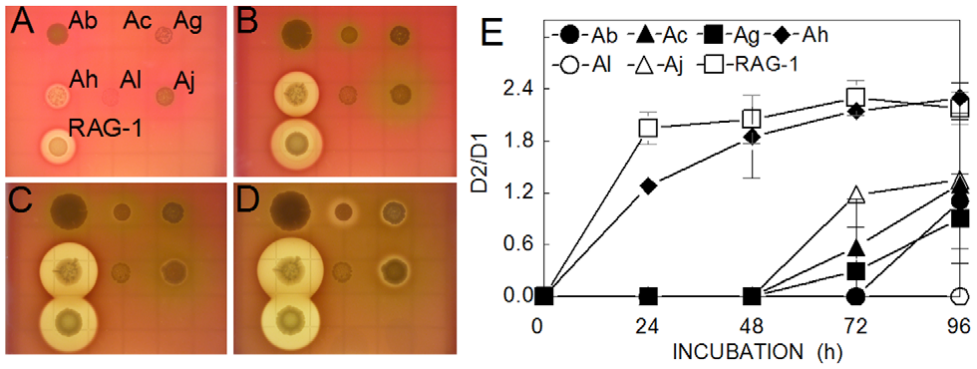
Expression of haemolytic activity. Sheep blood agar plates were inoculated with 10^3^ cfu/spot of each *Acinetobacter* species, and photographed at 24 h (A), 48 h (B), 72 h (C) and 97 h (D). Graph (E) shows ratios of the diameters of the colonies with their lytic zones (D2) divided by the diameters of the colonies only (D1). Data points are the means ± standard deviation from 4 separate plate assays.

The haemolytic potential of *Acinetobacter* strains was tested by quantifying the lysis of sheep red blood cells embedded in agar plates. All strains except Ac formed colonies within 24 h (Figure 1A), but only Ah and Av-RAG-1 produced large clearing zones, indicative of β-haemolysis comparable to that produced by the *B.cereus* positive control. With longer incubations, Ac, Ag, and Aj produced a weak response (α-haemolysis), beginning as faint grey-green rings (Figure 1B, C and D), gradually forming a thin clearing zone. No haemolytic activity (γ-haemolysis) was produced by Al colonies. The quantification of the haemolytic activity summarized in Figure 1E was derived from four replicate plates by comparing the diameters of the lytic zones (D2) with those of the corresponding colonies (D1). Further tests of the filtrates derived from 24 h cultures of these bacteria grown in nutrient broth confirmed that only Ah and Av-RAG-1 produced appreciable erythrocyte cytolysis (data not shown).

### Mammalian Cell Toxicity

**Figure 2:**
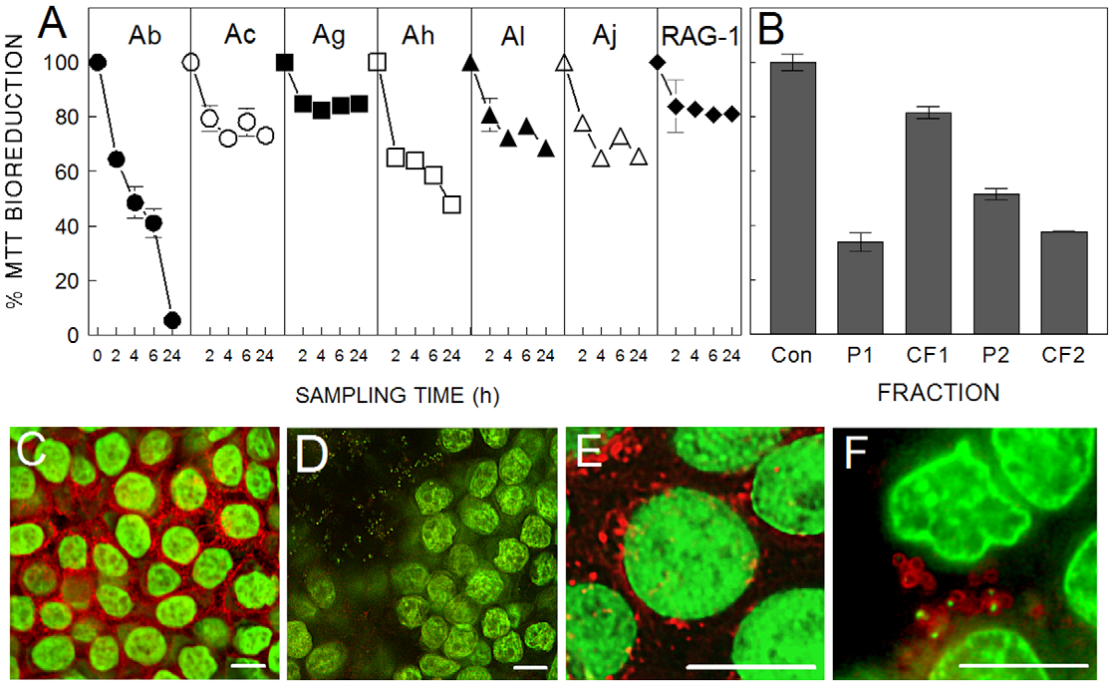
Toxicity of *Acinetobacter* towards human colonic epithelial cells. (A) HT29 cell monolayers were exposed to 10^6^ cfu/100 µL for up to 24 h and analyzed for bioreduction activity as described in Materials and Methods. (B) Bioreduction activity of HT29 cells following a 24-h exposure to Ab culture fractions in the presence of gentamicin. Bacterial cultures were centrifuged and separated into pellet (P1) and culture filtrate (CF1). The pellet was resuspended in fresh media, vigorously agitated and recentrifuged to yield a secondary pellet (P2) and filtrate (CF2). (C–F) Confocal micrographs of HT29 treated with PBS alone (C, E) or 10^6^ cfu/100 µL of Ab (D) or Ah (E) for 18 h, and then fixed and permeabilized as described in the Materials and Methods. Cells in C and D were stained with SYTOX™ green and rhodamine-conjugated phalloidin. Panels E and F were stained with SYTOX™ green and Texas Red-conjugated wheat germ agglutinin. Error bars in A and B represent the mean of three exposures ± standard deviation. Bars in C–F represent 10 µm.

Loss of metabolism, measured by bioreduction of MTT, and abnormal cell morphology were used as toxicity indicators for assessing HT29 and J774A.1 exposures to bacteria cells and/or their extracellular products. The data summarized in Figure 2A show that all strains caused at least 15–20% loss in total HT29 bioreduction capacity within two hours of exposure. At this stage there were no significant morphological changes other than decreased intracellular formazan staining. With longer exposure durations (18 h to 24 h), the most damaging strains were Ab and Ah, causing 95% and 50% drops in bioreduction per well, respectively.

Bacterial culture fractions of Ab were prepared to determine if the toxicant was associated with the bacterial cell or was an extracellular component. Cultures were separated into a bacterial cell pellet fraction (P1), and cell-free, culture filtrate fraction (CF1) by centrifugation and filtration. Figure 2B shows that when exposures were done with P1 in the presence of the antibiotic, gentamicin, HT29 metabolic loss and detachment were observed, as reflected by the 65% drop in bioreduction. In contrast, CF1 exposure resulted in only ∼20% bioreduction loss, and no cell detachment. If the bacterial cultures were agitated prior to filtration, the resultant filtrate (CF2) caused both toxicity and cell detachment, suggesting that the effectors were loosely associated with bacterial cell itself. Also, the toxic component of Ab was resistant to heat (100°C, 30 min).

Compared to Ab and Ah, Av-RAG-1 was least toxic in exposures to HT29 (Figure 2A). The diminished effects were attributed to the inability of Av-RAG-1 to grow in the presence of HT29 cells (Table 3). However, culture filtrates generated from Av-RAG-1 cultures in LB, were haemolytic and also caused ∼60% loss in bioreduction of HT29 cells (data not shown). This toxic component was released into the medium during culture of Av-RAG-1 without cell agitation prior to filtration.

Microscopic examination during Ab and Ah exposures showed that the damage could be attributed to a combination of diminished bioreduction capacity and numbers of attached HT29 cells. To detail this observation further, exposed cells were fixed and stained with dyes for the nucleus and fibrous actin (Figures 2C and 2D), or membrane polysaccharides (Figures 2E and 2F). Figures 2D and 2F show HT29 monolayers that were disrupted by Ab and Ah exposures. These exposures were all done at 18 h and not 24 h because very few HT29 remained attached at the latter time point. Ab caused HT29 monolayers to detach in entire sheets, whereas Ah caused individual cells to detach. In either case, inclusion of a protease inhibitor cocktail during the exposure prevented cell detachment. These inhibitors were neither toxic to HT29 nor to the bacteria as measured by MTT and XTT viability assays. However, microscopic examination of the wells supplemented with protease inhibitors revealed that Ab and Ah exposures still resulted in almost complete loss of HT29 intracellular bioreduction activity (ie., the cells exhibited no internal formazan deposits). Cells incubated with bacteria showed loss of actin-staining in Ab-exposed cells (Figure 2D) and wheat germ agglutinin-binding in Ah-exposed cells (Figure 2F) compared to corresponding unexposed controls (Figure 2C and 2E, respectively). Also apparent during microscopy, was bacterial adherence to the monolayer cells and surrounding well surfaces (Figures 2D and 2F). This binding was also observed in MTT assays, which indicated that formazan contributed by bacteria was highest in these exposures. Bacterial adherence was also demonstrated with fluorescence from wheat germ agglutinin–bound Ah (red stain in Figure 2F). The binding of lectins to the capsular polysaccharide of one strain of *A.venetianus* has been documented previously [61], but not for Av-RAG-1.

### Phagocytosis and Macrophage Bactericidal Activity

**Figure 3:**
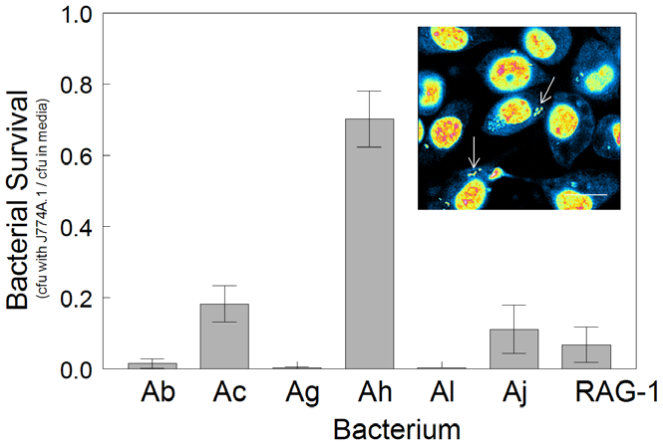
Phagocytosis and bactericidal activity of J774A.1 macrophage. Survival of bacteria was monitored by enumerating cfu's following 4-h incubation to 10^3^ cfu/100 µL with or without monolayers of J774A.1. Bacterial survival is expressed as the ratio cfu = s recovered from incubations with J774A.1 in mammalian culture medium (DMEM+supplements) and from mammalian culture medium alone. Inset: Phagocytosis of bacteria (Ah shown as example) was monitored by exposing J774A.1 monolayers to 10^6^ cfu/100 µL for 60 min, and then fixing and staining with SYTOX nucleic acid stain. Internalization was visualized using a confocal microscope, and artificial graded colour intensity (SYTOX intensity from blue to red) was used to highlight bacteria (arrows) within J774A.1. Data points represent the means of five separate experiments ∀ standard deviation. Bar represents 10 µm.

The capacity of J774A.1 to phagocytize bacteria and prevent infection *in vitro*, was examined using confocal microscopy, and coupled with tests for bacterial cell viability during exposure. Using the SYTOX™ stain, cells of each bacterial strain were initially detected on the apical surface of J774A.1 cells, but after ∼5 min they were seen to internalize and migrate towards the macrophage's basolateral surface. The photo inset of Figure 3 is typical of J774A.1 cells that have phagocytised Ah at the time of maximal uptake (60 min).

Experiments were done to determine whether certain strains had the ability to resist being killed by J774A.1. As shown in Figure 3 with 4-h exposure assays, the viability of most *Acinetobacter* strains was reduced by ≥80% when bacteria and macrophage were co-incubated, compared to bacteria in mammalian cell culture medium alone (or with HT29). The viability for most strains was lowest whether their cell numbers were low (10^3^ cfu/100 µL) or high (10^4^–10^6^ cfu/100 µL well). However, Ah consistently exhibited high viability, even at concentrations as low as 10–100 cfu/100 µL. At higher concentrations, recovered Ah cfu's were greater than the initial seeding concentration. Neither pre-activation of J774A.1 with IFN-γ, nor longer co-incubations of 7 to 24 h reduced Ah cfu numbers. Examination of time-course experiments by microscopy and colony enumerations revealed that the residual external bacteria were replicating, and not the internalized bacteria. This observation suggested that the surviving cfu = s measured in Figure 3 were primarily of external origin.

In other experiments bacteria were co-incubated with J774A.1 in wells with a 0.2 µm filter barrier between them. This exposure system resulted in no killing and demonstrated that phagocytosis or close interaction was needed for J774A.1-induced killing to occur. Parallel exposures conducted with HT29 epithelial cells did not result in lowered viability.

### Colonic Epithelial Cell Cytokine Production

**Figure 4:**
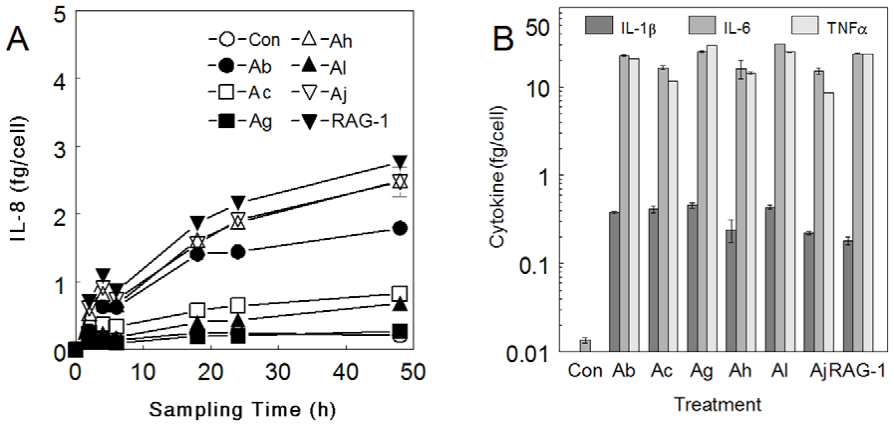
Mammalian cell inflammatory cytokine levels during exposure to test bacteria. HT29 (A) and J774A.1 (B) cells were exposed to 10^6^ cfu/100 µL test bacteria for various time intervals. Exposure supernatants were filtered and used in liquid array bead assays for IL-8 (A) or IL-1β, IL-6 and TNF-α (B). Experiments were reproduced twice using both the multiplex liquid bead assay and ELISA, both yielding results within 15% variability of each other. Data points are means of three exposures ∀ standard deviation.

Production of cytokines was monitored during exposures to determine if *Acinetobacter* strains could initiate epithelial inflammatory responses. Cytokine levels were quantified using multiplex liquid bead arrays for GM-CSF, IL-1β, MIP-1β, IL-6, IL-8, IL-12 and TNF-α, and verified with double antibody sandwich immunoassays. HT29 cells most consistently produced IL-8 during *Acinetobacter* exposures. Experiments with Ab and Ah indicated that in the absence of antibiotic, the build-up of IL-8 peaked at 6–8 h and was markedly reduced thereafter. The observed drop in levels was likely related to HT29 death and detachment, but also IL-8 degradation during bacterial growth. Inclusion of antibiotic throughout the exposure regime resulted in sustained levels of ILB8 (Figure 4A). Unexposed HT29 produced a low (constitutive) level of IL-8 in the supernatant. Exposure to *Acinetobacter* strains in the presence of antibiotic resulted in increased extracellular IL-8 levels which persisted for at least 48 h. The induced IL-8 production, measured by both multi-bead array and ELISA, could be divided into two statistically divisible groups (p<0.001 at 48 h). Strains of Ab, Ah, Aj and Av-RAG-1 induced between 1.7 and 3 fg IL-8 per HT29 cell, whereas Ac, Ag and Al generated levels of ≤1 fg/cell.

### Macrophage Cell Cytokine Production

Macrophage-like J774A.1 cells were tested for cytokine production in exposures similar to those for HT29 cells. The J774A.1 did not produce significant levels of neutrophil chemoattractants, such as KC, but instead produced IL-1β, IL-6 and TNF-α. These three cytokines are involved in the initiation of the acute phase response (APR). In 24-h exposures with gentamicin, all bacteria were strong inducers of the three cytokines, resulting in extracellular expression levels of 0.3 fg/cell or 0.75 ng/mL for IL-1β, 15 fg/cell or 38 ng/mL for IL-6 and 15 fg/cell or 38 ng/mL for TNF-α (Figure 4B). The cytokine levels were comparable to those produced by J774A.1 in control experiments using commercial preparations of LPS (0.1 to 1.0 ng/mL) from *Escherichia coli*, *Salmonella typhimurium* and *Serratia marcescens*.

### Presence of Virulence-related Genes

To determine if the strains differed in genes that have been reported to be overt toxins (*espA* (K1 capsular polysaccharide), *ompA* (outer membrane protein A)) [25] [47] [56], primers targeting those genes were made and used in PCR amplifications for amplicon size comparisons. In all cases, the appropriate sized amplicons were generated, suggesting that all the strains possessed similar gene segments.

### QRDR of *gyrA* and *parC* genes

**Figure 5:**
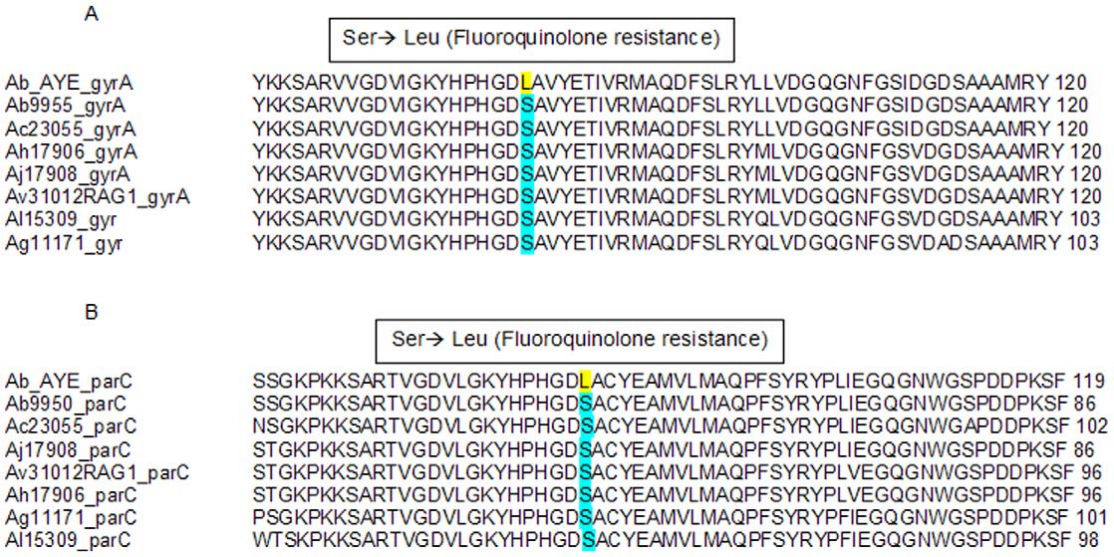
Alignment of Quinolone Resistance Determining Regions. ClustalW alignment of coding regions of *gyrA* (**A**) and *parC* (**B**) genes derived from test *Acinetobacter* strains compared to *Ab* AYE strain with known fluoroquinolone resistance mutations. The alignment demonstrates that the test strains lack a Ser-Leu transition necessary for fluoroquinolone resistance.

To determine if the strains differed in the QRDRs, PCR and nucleotide sequencing was carried out for the *parC* and *gyrA* genes. Sequences translated *in silico* were aligned with strain Ab AYE, known to have the amino acid substitution conferring resistance. Sequences from all strains lacked the leucine residues associated with fluoroquinoline resistance (Figures 5A for *gyrA* and Figure 5B for *parC*).

### Antibiotic Resistance

**Figure 6:**
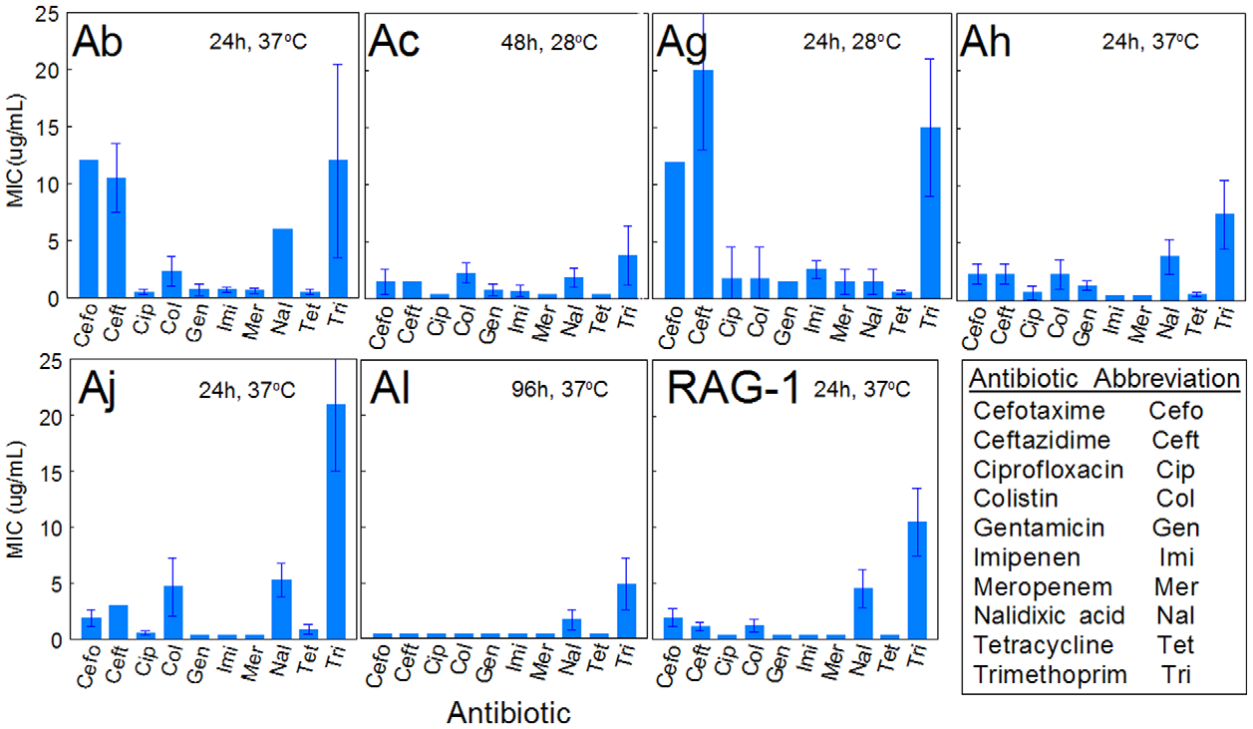
Antibiotic Susceptibility Assays. Antibiograms generated from growing each *Acinetobacter* strain in trypticase soy broth in the presence of different concentrations and classes of antibiotics for durations and temperatures indicated in the figure. The minimum inhibitory concentration (MIC) is the lowest concentration that is effective in preventing bacterial MTT bioreducton. Data are means of four experiments with vertical bars indicating standard deviation.

As a functional analysis of strain susceptibility towards a fluoroquinolone antibiotic and also several other antibiotic classes, bacterial MTT bioreduction activity towards a panel of antibiotics was tested. Figure 6 shows the resulting antibiograms, which clearly demonstrate that Ab was most resistant, albeit at low antibiotic concentration, to some test antibiotics. Ag and Aj also showed selective antibiotic resistance at low levels. Of these antibiotics, ciprofloxacin is a secondary fluoroquinolone. None of the strains showed high resistance to ciprofloxacin, which is consistent with the sequencing results in Figure 5 showing absence of mutations conveying fluoroquinolone resistance in genes, *parC* and *gyrA*.

## Discussion

This paper summarizes several *in vitro* bacterial and mammalian cell-based assays that permit differentiation between potentially hazardous or virulent *Acinetobacter* strains from relatively safe strains. The assays that were useful in discriminating the virulence of these bacterial strains are summarized in Table 4. As we have cautioned for other genera [62], broad species-level conclusions should not be generalized from the test strains examined here. The analyses conducted would need to be repeated for each strain under evaluation.

**Table 4:** Summary of Assays for Comparing Potential Virulence Characteristics of *Acinetobacter*.

Pathogenic Characteristic*	Ab	Ac	Ag	Ah	Aj	Al	Av-RAG-1
Bacterial Growth with Mammalian Cells	+	+	(+)	+	+	+	−
Haemolytic Activity	(+)	(+)	(+)	+	(+)	−	+
Mammalian Cell Detachment/Lysis	+	(+)	(+)	+	(+)	(+)	(+)
Bacterial Survival with J774A.1	−	(+)	−	+	(+)	−	(+)
HT29 Neutrophil Chemoattractant (IL-8)	(+)	−	−	+	+	−	+
Growth with Antibiotics**	+	−	+	(+)	+	(+)	(+)

+ signifies substantial growth or activity.

(+) signifies low level and/or delayed activity.

− signifies negligible growth or activity

* Note that tests for induction of J774A.1 pro-inflammatory cytokines (IL-1β, Il-6, TNF-α) and presence of known virulence gene segments (*OmpA*, *EpsA*) were excluded from the summary table since they failed to discriminate between bacterial strains.

** Growth for this purpose is defined as MTT bioreduction in the presence of at least 5 µg/mL antibiotic.

In initial experiments, we screened strains for their capacity to multiply or grow in mammalian cell culture medium at 37°C as a simple determinant of potential virulence. In previous studies of the *B.cereus*-group bacteria, XTT bioreduction was used to measure growth of each strain in culture media alone or in the presence of mammalian cells [53]. In preliminary studies we found that the rates of XTT reduction by the different *Acinetobacter* strains were similar in LB medium, but lower than the previously observed rates of *Bacillus spp by* ∼4–5 times. However, similar to the *Bacilli*, most of the *Acinetobacter* strains were unable to grow or grew poorly in mammalian cell culture medium at the lowest inoculation concentrations, unless supplemented with either mammalian cells or mammalian cell-conditioned medium (data not shown). The exception was the Ah strain, which grew well, with or without HT29 cells. In contrast, Av-RAG-1 did not grow well in mammalian culture medium even in the presence of HT29 or conditioned medium.

Another indicator of virulence is the production of toxic or lytic by-products by various strains. In experiments using the sheep blood agar plate assay, both Ah and Av-RAG-1 exhibited strong haemolytic activity (referred to as β-haemolysis) whereas all other strains exhibited weak (α- haemolysis) or no (γ-haemolysis) capacity. However, Av-RAG-1 was not able to grow in mammalian medium in absence or presence of HT29 like other strains. Our data match that of several other reports in that only Ah and Av-RAG-1 were haemolytic [63]–[66]. However, none of these studies described the haemolysis in a semi-quantitative manner, as we have done here. In another study, 526 *Acinetobacter* strains were tested for haemolytic activity with human, sheep and bovine erythrocytes, and only 16% of them exhibited β-haemolysis while the majority were α-haemolytic [51]. This study included 24 Ah strains of which 17 (70%) were β-haemolytic. Some of the strains used by Gospodarek *et al* [51] may have been misclassified or variable in expression of haemolytic acivity since an absence of haemolytic activity was seen in 20 nosocomial Ab isolates tested with rabbit erythrocytes [41]. Furthermore, Antunes and colleagues [50] have recently demonstrated that there is significant inter-strain variation in the haemolytic capacity of four different Ab isolates, which is also dependent on the source (horse or sheep) of blood used in the assays. The genetic factor(s) contributing to this haemolytic activity remains to be identified.

As a further screen for toxic and lytic products, we used two mammalian cell models. In exposures using the colonic epithelial HT29 bioreduction assay [37], toxicity ensued rapidly within two hours of exposure most notably for Ab and Ah compared to the other strains. Further studies showed that loss of mammalian cell bioreductive capacity may be underestimated due to bacterial adherence, which would have contributed to total MTT formazan measured per assay well. With crude fractionation, it was shown that the Ab-mediated detachment of HT29 monolayers was cell wall -associated, likely proteinaceous and could be the same component causing the loss of HT29 bioreduction capacity. In contrast, the toxic agent(s) of Ah was not necessarily associated with the bacterial cell, since bacteria-free culture filtrates also caused hemolysis and cytolysis. Furthermore, while Av-RAG-1 did not grow in presence or absence of HT29 it did produce an Ah-like, filterable, extracellular-cytotoxic activity (as well as haemolytic activity) when grown in LB broth. This activity was heat sensitive, whereas the Ab toxicant was not. [63]–[66]

Several studies have identified cytotoxic factors from various Ab strains. In particular, Ab produces outer membrane vesicles that contain a number of virulence-related factors [67]. One of these factors, Ab outer membrane protein A (AbOmpA), is suggested to play an important role in pathogenesis as evidenced by its pleiotropic effects. Purified AbOmpA was localized to the mitochondria of HEp-2 human laryngeal epithelial cells, and resulted in the release of pro-apoptotic molecules such as cytochrome c and apoptosis-inducing factor into the cytosol, caspase-3 activation, and DNA fragmentation [56]. None of our strains demonstrated a difference in the presence of genes for *ompA* or another reported virulence factor gene, *epsA*. Our studies used the same Ab strain as in previous work of Choi and colleagues [40], and yet we did not observe Ab invasion of HT29 colonic epithelial cells, supporting earlier observations that other cell types were not as permissive for invasion as respiratory epithelial cells. Comparison of wild-type and AbOmpA-negative isogenic mutants of Ab by Choi *et al* [40] demonstrated that AbOmpA was also involved in epithelial cell attachment, which we have observed during our studies.

Since there are concerns about antibiotic resistance of some *Acinetobacter* strains [68]–[70], we also tested inhibitory effects (MIC assay) of several antibiotics, and in particular, if the test strains carry known mutations in the constitutive genes *gyrA* and *parC* conferring fluoroquinolone resistance [57]–[58]. The genes for *gyrA* and *parC* were sequenced, and then translated and aligned *in silico*. None of the tested strains had the serine to leucine transition (*gyrA* codon 83 and/or *parC* codon 80 or 84) implicated in the fluoroquinolone resistance phenotype (Figures 5A and 5B). Functional susceptibility to fluoroquinolone was confirmed for all strains with an MIC assay. Furthermore, this assay demonstrated that the Ab strain exhibited the most resistance to the antibiotics in general, and the least effective antibiotics towards all strains were cefotaxime, ceftazidime, and trimethoprim. The MIC assay also revealed that none of the strains were resistant to the fluoroquinolone, ciprofloxacin.

As a measure of immune response, we screened for bacterial-induced release of select cytokines known to be associated with our mammalian cell models [39]. For HT29, all of the *Acinetobacter* strains induced IL-8 levels that were consistent with those recorded for other non-invasive bacteria [71]. This neutrophil chemoattractant has been used previously to differentiate between pathogens and generally harmless bacteria [71]–[74]. The present study showed that *Ab* induced less IL-8 production in HT29 cells, compared to *Aj*, *Ah*, and *RAG-1*. Our results agree with a recent study by de Breij and colleagues [1] who reported that human epithelial cells produced less IL-8 in response to *A. baumannii* strains than to *A. junii* strains. They also suggested that *A. baumannii* appeared to be more virulent compared to the other strains, since infection caused by *A. baumannii* was associated with reduced capacity of bacterial elimination from the host. Furthermore, we agree with their comment that outgrowth may have caused increased elevation of the cytokines, as we see substantial growth in the presence of HT29 cells over a 24-h period (Table 3). However, our cytokine experiments were done in the presence of antibiotic to avoid the effect of differential growth on cytokine production, but also to limit any cytotoxicity associated with proliferating bacteria.

In contrast to the high levels of HT29 IL-8 release, J774A.1 released several pro-inflammatory cytokines, but none which were useful for differentiating between the strains tested. The similarity in the levels of J774A.1 cytokines, regardless of test bacterial strain, is consistent with known mechanisms of macrophage receptor-mediated recognition of bacterial pathogens. That is, cytokine induction is largely a result of pathogen-associated molecular patterns (PAMPs) made up of well-conserved microbial surface moieties, such as LPS or peptidoglycan (see reviews: [75]–[78].

Huttunen and colleagues [79] have used IL-1β, IL-6, and TNF-α expression from RAW264.7 macrophage as toxicological indicators of hazard from indoor microbial sources. In their study, doses up to 10^7^ cfu/mL (equivalent to our 10^6^ cfu/100 µL well), caused different levels of acute phase response (APR) cytokines depending on the bacterial strain tested. *Bacillus cereus* was least inducing (eg. 3.1 ng/mL TNFα), *Streptomyces californicus* caused an intermediate level (6.1 ng/mL TNF-α) and *Pseudomonas fluorescens* was marginally better (6.9 ng/mL TNF-α). In preliminary comparative tests (data not shown), we found that when the macrophage-like cell line RAW264.7 was exposed to *Acinetobacter* strains, it expressed APR cytokines at similar levels as J774A.1. However, those levels were at least 5-fold higher than those observed by Huttunen and colleagues with the RAW264.7 cells. The difference is most probably due to macrophage densities during exposure, underlining the importance of expressing cytokine expression data on a per-cell basis. In addition, bacterial species or strain differences likely play a role in the differential APR cytokine expression levels observed here. The lack of a difference we observed with our test system suggests that APR cytokine expression is not discriminatory at the species level for *Acinetobacter*, but may be a more relevant indicator when comparing between genera.

Our data with J774A.1 cells clearly demonstrate that Ah is the most resistant to macrophage-induced inactivation. Unlike other claims that addition of 0.004–0.1% Triton X-100 facilitates release of internalized bacteria and improves colony recovery [80], we found that the detergent was ineffective between 0.004–0.05% in lysing J774A.1 cells, and inhibited bacterial growth above 0.05%.

In conclusion, this comparative study shows the utility of *in vitro* assays and molecular probes for assessing for growth, toxicity, infectivity and potential immune responses to aid in screening virulence potential of select *Acinetobacter* species or strains. Collectively, these analyses have demonstrated that the Ab and Ah strains used here are likely to be the most hazardous if used in a biotech process involving large scale release. In contrast, the environmental strain Ag was relatively benign for growth in mammalian environments, toxicity and intracellular infectivity. Further, the Av-RAG-1 bioremediation strain was relatively non-infectious, but it had strong haemolytic capacity and induced production of inflammatory chemokines. These observations suggest that Av-RAG-1 should be investigated in more detail for potential clinical effects prior to environmental use as an industrial-scale bioremediation agent. Furthermore, our results suggest that ompA and epsA are not the primary virulence factors for these test stains. The next phase of our research will focus on confirming these *in vitro* observations using analogous *in vivo* murine exposure scenarios as recently reported for screening *Bacillus* organisms [39].
